# Emergency department-based, nurse-initiated, serious illness conversation intervention for older adults: a protocol for a randomized controlled trial

**DOI:** 10.1186/s13063-022-06797-6

**Published:** 2022-10-09

**Authors:** Thidathit Prachanukool, Susan D. Block, Donna Berry, Rachel S. Lee, Sarah Rossmassler, Mohammad A. Hasdianda, Wei Wang, Rebecca Sudore, Mara A. Schonberg, James A. Tulsky, Kei Ouchi

**Affiliations:** 1 Department of Emergency Medicine, Brigham and Women’s Hospital, Boston, MA USA; 2 Harvard Medical School, Boston, MA USA; 3 Department of Emergency Medicine, Faculty of Medicine Ramathibodi Hospital, Mahidol University, Bangkok, Thailand; 4 Department of Psychosocial Oncology and Palliative Care, Dana-Farber Cancer Institute, Boston, MA USA; 5 Division of Palliative Medicine, Department of Medicine, Brigham and Women’s Hospital, Boston, MA USA; 6 Department of Biobehavioral Nursing and Health Informatics, University of Washington School of Nursing, Seattle, WA USA; 7 Department of Nursing, MGH Institute on Health Professions, Boston, MA USA; 8 Division of Geriatrics and Palliative Care, Baystate Medical Center, Springfield, MA USA; 9 Division of Circadian and Sleep Disorders, Departments of Medicine and Neurology, Brigham and Women’s Hospital, Boston, MA USA; 10 Division of Geriatrics, Department of Medicine, University of California San Francisco, San Francisco, CA USA; 11 Department of Medicine, Beth Israel Deaconess Medical Center, Boston, MA USA

**Keywords:** Geriatrics, Emergency medicine, Palliative care, Motivational interviewing

## Abstract

**Background:**

Visits to the emergency department (ED) are inflection points in patients’ illness trajectories and are an underutilized setting to engage seriously ill patients in conversations about their goals of care. We developed an intervention (*ED GOAL*) that primes seriously ill patients to discuss their goals of care with their outpatient clinicians after leaving the ED. The aims of this study are (i) to test the impact of *ED GOAL* administered by trained nurses on self-reported, advance care planning (ACP) engagement after leaving the ED and (ii) to evaluate whether *ED GOAL* increases self-reported completion of serious illness conversation and other patient-centered outcomes.

**Methods:**

This is a two-armed, parallel-design, single-blinded, randomized controlled trial of 120 seriously ill older adults in two academic and one community EDs in Boston, MA. Participants are English-speaking adults 50 years and older with a serious life-limiting illness with a recent ED visit. Patients with a valid MOLST (medical order for life-sustaining treatment) form or other documented goals of care within the last 3 months are excluded. We enroll the caregivers of patients with cognitive impairment. Patients are assigned to the intervention or control group using block randomization. A blinded research team member will perform outcome assessments. We will assess (i) changes in ACP engagement within 6 months and (ii) qualitative assessments of the effect of *ED GOAL*.

**Discussion:**

In seriously ill older adults arriving in the ED, this randomized controlled trial will test the effects of *ED GOAL* on patients’ self-reported ACP engagement, EMR documentation of new serious illness conversations, and improving patient-centered outcomes.

**Trial registration:**

ClinicalTrials.gov identifier: NCT05209880

**Supplementary Information:**

The online version contains supplementary material available at 10.1186/s13063-022-06797-6.

## Background

Serious illness conversations are discussions between patients with advanced illness and their clinicians that focus on their values, goals, and priorities related to their health care [1]. As part of a comprehensive care plan, serious illness conversations can lead to well-informed shared decision making and improved quality of life at the end of life [2]. For seriously ill older adults (expected prognosis of < 1 year), these conversations may be associated with lower rates of in-hospital death, less aggressive medical care at the end of life, earlier hospice referrals, increased peacefulness, and a greater likelihood to have end-of-life wishes known and followed [2–9]. Furthermore, one study reported that patients who had documented serious illness conversations could reduce expenses by 36%, with each patient saving $1041 on average during their last week of life [10]. Experts recognize that earlier serious illness conversations may be among the keys to “bending the cost curve” for health care [11]. Yet, only 37% of seriously ill older adults have these conversations with their physicians [2], on average 33 days before death [12].

Emergency departments (ED) may serve as an ideal setting to engage seriously ill, yet clinically stable, older adults who may benefit from serious illness conversations. During the last 6 months of life, 75% of older adults visit the ED [13]. ED visits are inflection points in these patients’ illness trajectories, signaling a more rapid rate of decline [14–16]. Furthermore, seriously ill older adults have a 24–48% mortality following these ED visits [17–19]. More than 70% of these patients express priorities focused on comfort and quality of life rather than life extension [20], yet a systematic review revealed that 56–99% do not possess advance directives in the ED [21], and many are at risk of receiving care that does not align with their goals [22]. To leverage this opportune moment in the ED, we developed and tested a behavioral intervention to engage seriously ill older adults in serious illness conversations (*ED GOAL*) to overcome the known barriers to serious illness conversations in this setting (e.g., time constraints, limited privacy, uncertainty in patients’ awareness of their illness) [23]. Guided by the Social Cognitive Theory [24] and modeled from previously successful ED-based behavioral interventions [25–30] using the Transtheoretical Model [31], *ED GOAL* consists of a short, motivational interview that aims to prime patients to discuss their goals of care with their outpatient clinicians rather than triggering a more time-consuming, sensitive conversation in the time-pressured ED. In a small pilot of ED physicians (and advance practice clinicians) speaking with 23 seriously ill older adults, 82% found *ED GOAL* acceptable and stated that it helped them engage in conversations about their goals for future care with their outpatient clinicians. Yet, emergency physicians were often interrupted; thus, limiting *ED GOAL*’s implementation [32]. ED nurses suggested that a specially-trained, nurse consultation model would result in improved efficacy because motivational interviewing is within their scope of practice [33–38]. In a feasibility study of trained study nurses who enrolled and conducted the intervention with 76 patients, the self-reported readiness to engage with outpatient physicians increased from 2.8 to 3.3 on a 5-point Likert scale (*p* = 0.008), and 16% of the patients reported that they talked to their primary outpatient clinician about their future care at 1 month after the intervention. Most participants (62%) reported that after *ED GOAL* they felt “completely” heard and understood by the study nurse about what they would want in medical care if they were to get sicker, compared to only 15% who felt this way with their outpatient clinicians. In addition, 16%, 25%, and 33% of participants had a new documentation of serious illness conversations with their outpatient clinicians at 1, 3, and 6 months, respectively, suggesting that *ED GOAL* successfully led to patient-clinician communication about goals of care in the outpatient setting. (ClinicalTrials.gov identifier NCT04730986, under peer review).

Despite these promising findings from observational studies, the efficacy of *ED GOAL* has not been established in a randomized study. Therefore, in this study protocol, we describe a two-armed, parallel, single-blinded, randomized controlled trial of seriously ill older adults in three EDs in Boston, MA. We will compare the *ED GOAL* intervention group to a control group receiving usual care. The objectives of this study are (i) to test, in a randomized controlled design, the effect of *ED GOAL* administered by trained nurses on patient- and caregiver-reported ACP engagement one month after leaving the ED (primary outcome) and (ii) to evaluate the impact of *ED GOAL* on self-reported completion of serious illness conversations, new, documented serious illness conversations in the electronic medical records (EMR), quality of communication, health care utilization, and survival.

## Methods

### Design

This is a two-armed, paralleled-design, single-blinded, superiority randomized controlled trial of 120 seriously ill older adults in two academic and one community EDs in Boston, MA. The institutional review board at Mass General Brigham approved all study procedures (institutional review board protocol #2021P003093), and all participants will provide informed consent.

### Setting

All three participating centers are located in Boston, Massachusetts. Two quaternary care academic medical centers are 1059-bed hospital with 100,000 annual ED visits and 793-bed hospital with 57,000 annual ED visits, respectively. One community hospital has 171 beds and 30,000 annual ED visits. All EDs provide clinical care 24 h per day, 7 days per week.

### Participants (Table 1)

#### Inclusion criteria

Patients eligible for the study include English-speaking adults 50 years and older with serious life-limiting illness (metastatic cancer, oxygen-dependent chronic obstructive lung disease, chronic kidney disease on dialysis, New York Heart Association class III or IV heart failure, treating ED clinician “would not be surprised if the patient died in the next 12 months”). Patients with non-metastatic cancer, chronic obstructive lung disease not on home oxygen, chronic kidney disease not on dialysis, or New York Heart Association class I or II heart failure are also eligible if they were hospitalized in the last 12 months for their serious illness and/or deemed clinically appropriate by ED clinicians. The patients who meet these criteria and have mild cognitive impairment or mild dementia are eligible to participate with their caregiver as their study partner. For patients with moderate or severe dementia, the caregivers are the sole subjects for the study. The caregivers are defined as health care proxy or next of kin (when health care proxy has not been appointed).

**Table 1:** Eligibility criteria

Inclusion	Exclusion
1. ≥ 50 years of age AND ≥ 1 serious illness* *OR* ED clinician would not be surprised if patient died in the next 12 months (a validated prognostic sign) [39, 40] 2. English-speaking 3. Capacity to consent a. Patient with mild cognitive impairment or mild dementia with capacity to consent (requires a caregiver/study partner to enroll) b. Caregiver of patient with moderate/severe dementia with capacity to consent	1. Acute physical or emotional distress 2. Determined by treating or study clinician not to be appropriate 3. Clearly documented goals for medical care** (Unless the treating or study clinician recommends that the intervention is clinically indicated) 4. Delirium (assessed using 3D-CAM) 5. Already enrolled in this study 6. Unable/unwilling to schedule the follow-ups on the calendar 7. Receive both the outpatient care for serious illness and primary care outside of the MGB system
*NYHA stage III/IV congestive heart failure, chronic obstructive lung disease on home oxygen, chronic kidney disease on dialysis, or metastatic solid tumor cancer. In addition, patients with NYHA stage I/II congestive heart failure, chronic obstructive lung disease not on home oxygen, chronic kidney disease not on dialysis will be included if recent hospitalization in the last 12 months exists	**e.g., MOLST, medical order for life-sustaining treatment, documented serious illness conversations in clinician notes within the last 3 months, etc.

#### Exclusion criteria

Patients are excluded if they have clearly documented goals for medical care, including a serious illness conversation in the recent 3 months or a presenting medical order for life-sustaining treatment (MOLST) in the EMR. We also exclude patients who are determined by the treating ED or outpatient clinician not to be appropriate, if they are unable to schedule the enrollment due to logistical challenges, have delirium, have both primary and specialty care for their serious illness outside of our health system, or have been enrolled in our previous feasibility study.

#### Recruitment (Fig. 1)

The study was introduced to the ED attending physicians during a faculty meeting and via email before the study initiation. To identify potential subjects consecutively, our research team reviews the EMR of the daily patient lists in the ED seven days per week, 24 h per day. Our team contacts eligible patients during their ED visit or within 10 days of their ED discharge. When COVID restrictions prevented us from conducting *ED GOAL* physically in the ED, we demonstrated the feasibility of conducting our study virtually using the phone or Zoom. Enrollments occur either physically or virtually by letters, emails, and phone calls to schedule at patients’ earliest convenience. We could enroll in the ED, during a hospital stay, or after discharge.

**Fig. 1:**
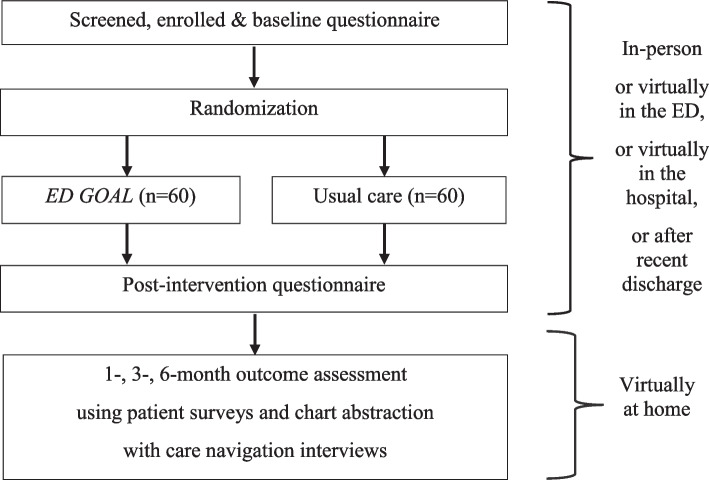
Study schema

When the research team identifies a potentially eligible patient, the patient will be interviewed to confirm eligibility. The study clinician will explain the study (supplemental file 1), obtain verbal informed consent from participants at the time of enrollment, and then screen them for delirium and cognitive impairment screening. The informed consent will be attained directly from patients with normal cognition, from patients and caregivers for patients with mild cognitive impairment or mild dementia, or from caregivers only for patients with moderate or severe dementia. Participants will be offered a $48 gift card for participation. Multiple forms of contact will be used to minimize loss to follow-ups. To comply with the NIH recommendations for clinical trials, once we approach 50% of the enrollment target goals for each arm, we will begin to preferentially recruit minority and under-represented populations if they are < 20% of the enrolled subjects to ensure that our study adequately represents the study population.

#### Delirium and cognitive impairment screening

The study clinician begins the enrollment by administering a three-minute diagnostic assessment, the Confusion Assessment Method (3D-CAM) [41] screening, to assess for delirium if patients are enrolled in the ED. Patients who are enrolled virtually after their ED/hospital visit will skip this step, assuming they are without delirium outside of the hospital. If the patient is found to not have delirium, the study clinician will then conduct the cognitive impairment screening. For patients who do not have any documentation of cognitive impairment or dementia in the EMR, the study clinician administers Mini-Cog© [42]. Patients who do not pass this screening (score < 3) are required to have a caregiver as their study partner to enroll. The study clinician will proceed with the Quick Dementia Rating System (QDRS) [43] screening with the caregiver. If the QDRS score indicates mild cognitive impairment or mild dementia (2–12), the enrolling subjects are both the patient and caregiver. If the QDRS score indicates moderate or severe dementia (13–30), the enrolling subject is the caregiver only. For patients who have documentation of cognitive impairment or dementia in the EMR, the study clinician skips the Mini-Cog© and simply starts with QDRS with the caregiver to determine the enrolling subject.

### Intervention

#### Randomization and blinding

After the subject enrolls, the trained RA administers the baseline assessment surveys for participants in both the control and intervention arms. The RA enrolling the patient will only accesses the sequence generation tool after the participant consents to the study. The RA then notifies the study clinician privately of the allocation and continues the enrollment, thereby keeping the patient blinded to the treatment allocation. The allocation sequence and computer tool are generated by a biostatistician who is not part of the research procedures. The list linking patient names and group assignments is stored on a secure network computer under password protection. We used computer-generated block randomization of four algorithm. The blinded RAs who perform the follow-up outcome assessments do not have access to the randomization tool or any baseline data that indicates to which group the subject has been assigned. The outcome assessors and data analysists will be blinded to the intervention assignments.

#### Intervention arm

For patients assigned to the intervention arm, *ED GOAL* [32, 44, 45] will be conducted by trained research nurses in the ED. The clinician training included a 1-h didactic on research methodologies, motivational interviewing, and serious illness communication skills, followed by a 4-h communication training with trained actors. The training has been described previously [32, 44, 45]. *ED GOAL* will take approximately 13 to 15 min. Following *ED GOAL*, the participants in the intervention arm will be asked to rate the quality of communication by the study clinician and how well they felt heard and understood about what they would want in medical care if they were to get sicker. The study clinician will document what participants shared regarding their values and preferences if they were to get sicker in the EMR, communicate these findings to patients’ outpatient clinicians with patients’ permission, and provide a handout designed to encourage further serious illness conversations with their clinicians and family [46]. The values and preferences categories use patient-tested language that have been studied rigorously in prior studies [47–49]. The study team schedules follow-up appointments with patients’ outpatient clinicians whenever feasible and desired by participants.

The intervention fidelity will be measured for all enrollments to ensure that study clinicians deliver the intervention consistently. Clinicians who do not meet high fidelity, defined as mean adherence of > 70% of the components on the fidelity checklist [45, 50], will be retrained by the PI. Additionally, a doctoral-level nurse champion with a specialty-level certification in palliative care (SR) will review every patient enrollment and provide coaching to the trained nurses.

#### Usual care

If assigned to the control arm, the participants will not go through *ED GOAL* and receive care as usual. After the consent, the baseline and follow-up assessments will take place similarly to the intervention arm participants. They will not receive any handouts and their clinicians will not be notified.

### Outcome measures (Tables 2 and 3)

Our primary outcome is the change in self-reported ACP engagement to talk to outpatient clinicians about their values and preferences (item #3 in the validated, 4-item ACP Engagement Survey [51, 52], “How ready are you to talk to your doctor about the kind of medical care you would want if you were very sick or near the end of life?”) 1 month after the ED visit. Our secondary outcomes are other 3-items on the ACP Engagement Survey (a 5-point Likert scale, “I have never thought about it (1)” to “I have already done it (5)”) to measure participants’ readiness to (1) appoint a healthcare proxy; (2) discuss goals of care with their healthcare proxy; and (3) sign official documents to put their wishes in writing.), new EMR documentation of serious illness conversations, self-reported occurrence of serious illness conversations by patients and/or caregivers evaluated using a previously validated dichotomous survey item [46, 53], heard and understood survey [54] modified to fit the context of serious illness conversations (“How well they feel heard and understood by their primary outpatient clinician about medical care they would want if they were to get sicker?” a 5-point Likert scale “Not at all (1)” to “Completely (5)”), quality of communication survey [55] (4 end-of-life items selected a priori, a 10-point Likert scale “Worst you can imagine (0)” to “Best you can imagine (10)”), and qualitative interviews conducted with patients in the intervention arm at 1, 3, and 6 months from baseline. We will also look at healthcare utilization (i.e., ED visits and hospitalizations) up to 12 months pre- and post-enrollment. Both independent and dependent variables are collected via an interview with the patient or obtained from the EMR when appropriate.

**Table 2:** Independent variables assessed at the time of enrollment

Variables	Measurements/instruments	Sources
Treatment group	*ED GOAL* or usual care	Research coordinator
Age	Years	Medical record
Gender	Male or female	Medical record
Race/ethnicity	White, black, Asian, Hispanic, native Americans, others	Patient interview
Serious illness diagnosis	Illness type and stage	Medical record
Delirium status	A three-minute diagnostic assessment-Confusion Assessment Method (3D-CAM) [41]	Patient interview
Cognitive impairment status	Mini-Cog© [42]	Patient interview
Dementia status	Quick Dementia Rating System (QDRS) [43]	Patient interview
Primary caregiver	Relationship	Patient interview
Advance directives	Living will, health care proxy, medical order for life sustaining treatment	Medical record
Previously documented serious illness conversations	Patients’ stated hopes, worries, trade-offs, minimal quality of life, states worse than dying, preferred place of death, and preferences for cardiopulmonary resuscitation.	Medical record

**Table 3:** Dependent variables

Variables	Measurements/instruments	Sources
Primary		
The changes in the self-reported ACP engagement to discuss goals for end-of-life care with primary doctor	Item #3 of the 4-items ACP Engagement Survey, 5-point Likert scale [51]	Patient interview
Secondary		
The changes in the self-reported ACP engagement to appoint a healthcare proxy	Item #1 of the 4-items ACP Engagement Survey, 5-point Likert scale [51]	Patient interview
The changes in the self-reported ACP engagement to discuss goals for end-of-life care with the healthcare proxy	Item #2 of the 4-items ACP Engagement Survey, 5-point Likert scale [51]	Patient interview
The changes in the self-reported ACP engagement to sign official documents of the wishes for end-of-life care	Item #4 of the 4-items ACP Engagement Survey, 5-point Likert scale [51]	Patient interview
The new EMR documentation of serious illness conversations and advance directives within 6 months of the intervention	The dichotomous item	Medical record
The self-reported occurrence of serious illness conversations by patients and/or caregivers	The dichotomous item [46, 53]	Patient interview
The heard and understood survey modified for end-of-life care	2-item, 5-point Likert scale survey [54] (“How well they feel heard and understood by their primary outpatient clinician about medical care they would want if they were to get sicker?”)	Patient interview
The quality of communication of the serious illness conversation with the primary outpatient clinician	4 end-of-life items selected a priori, a 10-point Likert scale survey [55] “Worst you can imagine (0)” to “Best you can imagine (10)”	Patient interview
How the intervention may have affected conversations and actions surrounding ACP process if participant self-report having spoken to the primary outpatient clinicians	A brief qualitative interview	Patient interviews
The barriers to proceeding with serious illness conversations if the participant has not spoken to the primary outpatient clinician at six-month follow-up	A brief qualitative interview	Patient interviews
The healthcare utilization (ED visits and hospitalizations) within twelve months pre- and post-intervention	Count	Medical record

*ACP* advance care planning, *ED* emergency department

### Data collection and management

The research team is trained to understand that data collection never interferes with medical care and the interview is stopped for any reason related to patient care. The RAs administer the baseline survey electronically on a tablet computer using REDCap (Research Electronic Data Capture), a secured web-based application designed to support data capture for research studies [56]. REDCap surveys will only be created on encrypted, password protected computers that only the study staff can access. We will also use Dropbox Business and LabArchives, which are fully encrypted and approved by our hospital’s policies. To ensure the scientific dissemination and transparency, de-identified data required to replicate our study can be supplied upon request with data usage agreements. An emergency unblinding is not planned and a data safety monitoring board will not be required, as this is a minimal risk study and does not meet the criteria for an NIH-defined phase III trial. We do not anticipate any major adverse events from this minimal risk trial. Therefore, no compensation is planned for those who suffer harm from trial participation. The research team will report any adverse events immediately to the PI, who is responsible for safety monitoring of the research to the IRB and to the NIH institute. Our IRB reviews the conducts of all studies annually during our continuing review process. In addition, the institutional research compliance office conducts random audits of all studies throughout the years. Our study team reviews the trial procedures and conducts weekly with the PI, who is solely responsible for the conduct of the trial.

The unblinded RA will conduct the baseline assessments. For the follow-up assessments, the blinded RAs will conduct the assessments at 1, 3, and 6 months. Follow-up calls will be conducted as soon as the contact window (± 10 days) is open. A minimum of three attempts will be made to reach patients for promote and complete the follow-up assessments. If participants report having spoken to their outpatient clinicians about their values and preferences [46, 53], the blinded RA will then (i) conduct a validated survey to measure the quality of communication of the serious illness conversation with the primary outpatient clinician [55], (ii) ask the validated heard and understood question modified for the context of end-of-life care (“how well they feel heard and understood by their primary outpatient clinician about medical care they would want if they were to get sicker?”) [54], and (iii) conduct a brief qualitative interview. The goal of the interview is to capture how the intervention may have affected conversations and actions surrounding ACP process. If patients report having spoken to their outpatient clinician about their values and preferences after the enrollment and these additional questions have been answered, we will not conduct any subsequent follow-up calls. If participants have not spoken to their primary outpatient clinicians by the final follow-up call at 6 months, the blinded RA will conduct the qualitative interview then asking about barriers to proceeding with serious illness conversations.

For the EMR outcomes using the standardized methods [57], trained RAs will complete chart abstraction using a codebook to search for new serious illness conversation documentation and advance directives (e.g., MOLST, HCP) within 6 months of *ED GOAL* for all enrolled patients. We will also look for ED visits and hospitalizations within 12 months pre- and post-intervention.

### Analysis

We plan to enroll 120 patients, 60 in each arm. Our prior pilot study demonstrated a mean patient-reported ACP engagement increased from 3.8 to 4.3 on a 5-point Likert scale, corresponding to a moderate effect size of 0.50 [58]. A conservative estimate of effect size is 0.25, and we also expect that 10% of enrolled patients will die before completing all follow-ups. With a sample size of 60 patients per group, we would have 90% power to detect the difference using a 2-sided Fisher’s exact test (alpha = 0.05).

The mean changes in self-reported ACP engagement will be compared by study arms to estimate effect sizes. Within arms, we will use a one-sample binomial exact test of proportions for categorical outcomes (e.g., EMR documentation), and Wilcoxon signed ranks test for ordinal outcomes (e.g., ACP Engagement Survey) at baseline and at 1, 3, and 6 months after the intervention. A *p*-value of 0.05 will be the significance threshold. For qualitative interviews conducted with patients who have received the intervention, we will record, analyze, and professionally transcribe to identify any themes surrounding qualitative benefits (e.g., how the interview helped serious illness conversations with loved ones and clinicians) and barriers in ACP navigation (e.g., how attempted conversations were unsuccessful). Discrepancies that arise will be resolved by consensus, and thematic analysis will be performed.

For additional analyses, we suspect that patients who are hospitalized have a propensity to have more serious illness conversation documentation than those who are not, so we will use logistic regression in subgroup analysis for serious illness conversation documentation. We will also perform sensitivity analyses for the survey participant types (e.g., patients vs. caregivers). We will compare patient demographics by study arm to assess randomization using bivariable analyses (*t*-test for continuous variables and chi-square for categorical variables). Moreover, we will conduct a secondary analysis using linear mixed models at baseline and the follow-ups to address missing data if missing is > 5% of the data.

## Discussion

We describe our planned protocol for a clinical trial to determine the efficacy of our serious illness conversation intervention, *ED GOAL*, on self-reported ACP engagement and improving patient-reported ACP outcomes, health care utilization, and survival for seriously ill older adults presenting to the ED. This trial with both academic and community EDs will provide the efficacy estimate of our intervention for a larger, multi-center trial.

Interventions that are designed to improve serious illness communication between patients and clinicians have demonstrated in randomized trials to lead to more frequent, earlier, and better serious illness conversations and to greater documentations of ACP and goals of care discussions in the EMR [46, 49], which may also lead to reduced anxiety and depression in these patients [59]. At the same time, patients who receive these interventions may also have lower health care cost [60], though results are not readily replicable. Though these results are encouraging, no such trials have been conducted in the ED setting to facilitate serious illness conversations. To our knowledge, this is the first clinical trial to evaluate the efficacy of similar serious illness conversation intervention in the ED settings, which may have different effects than prior trials.

Our trial design has several key components built in to advance the field of palliative care research in the ED. Recruitment of seriously ill older adults during/shortly after the acute care visit is clinically timely to engage them in serious illness conversations. Given that serious ill older adults visit the ED in critical times of their illness trajectories, the potential exist to systemically capture all such patients at risk of inadequate ACP leading to end-of-life care misaligned to their values and preferences before their death. At the same time, the complexity of the care environment mentioned above limits robust research in the integration of palliative care and emergency medicine. To overcome this barrier, we chose to deliver our serious illness conversation intervention virtually from a centralized team who can deliver it with high intervention fidelity, having future dissemination in mind. We also added care coordination components to communicate the values and preferences that our participants shared with their primary outpatient clinicians. To maximize the chances of success in participant engagement, we also included a patient-facing handout modeled from a previously successful intervention to stimulate serious illness conversations [46, 48]. With these innovations, we hypothesize that our ED-initiated serious illness conversation intervention will increase self-reported ACP engagement and other important patient-centered outcomes in seriously ill older adults. The ultimate goal is to establish *ED GOAL* as a national standard of care to help all seriously ill older adults to receive serious illness conversations at the most critical times of their lives. We aim to expand the scope of ED-based care from acute, disease-oriented care (e.g., gunshot wounds) to include patient-centered care (e.g., value-based, end-of-life care) for seriously ill older adults by integrating geriatrics and palliative medicine principles.

## Trial status

Registration information is available at ClinicalTrials.gov (Identifier: NCT05209880). Recruitment began March 2022, and the anticipated completion date is March 2023. All results will be disseminated on ClinicalTrials.gov and publications. The datasets analyzed during the current study and statistical code are available from the corresponding author on reasonable request, as is the full protocol.

## Supplementary Information

**Additional file 1: Supplemental file 1.** Study information sheet for patients.

## Abbreviations

ACP: Advance care planning
ED: Emergency department
EMR: Electronic medical record
HCP: Healthcare proxy
IRB: Institutional review board
MOLST: Medical Orders for Life-Sustaining Treatment
PI: Principal investigator
RA: Research assistant
