# Supplementary material for: Emergency department-based, nurse-initiated, serious illness conversation intervention for older adults: a protocol for a randomized controlled trial
Source: Trials. 2022 Oct 9;23:866. doi: 10.1186/s13063-022-06797-6 (PMC9549655; doi:10.1186/s13063-022-06797-6)
Supplement: Supplementary file 1 — Additional file 1: Supplemental file 1. Study information sheet for patients. [file 13063_2022_6797_MOESM1_ESM.docx]

**Supplemental file 1. Study Information Sheet for Patients**

**Study Title:** An advance care planning intervention in the emergency department: a randomized controlled trial

**Principal Investigator (PI):** Kei Ouchi, MD, MPH

**Purpose of the Research:** Advance care planning (ACP) is making decisions about the medical care you would want to receive when you face a medical crisis in the future. These are your decisions to make based on your personal values, preferences, and discussions with your loved ones. Our research hopes to help people like you who are in emergency rooms begin to think about this planning.

**Funder of the Research:** Cambia Health Foundation/National Institutes of Health

**How we obtained your name and contact information:** We asked your Brigham and Women’s Hospital emergency room clinicians to identify you.

**Why are we asking you to participate?**

We are asking you to participate because we want to understand whether our trained clinicians can help you talk to your doctors about ACP after leaving the emergency department.

**How many people are anticipated to participate?**

We are asking about 120 patients to take part in this study.

**How long it will take to complete the study and what does it entail?**

The study will take place in the emergency room or over the phone/hospital-approved Zoom if COVID-19 protocols prohibit us from interacting in-person or if you prefer to do this study after your emergency room visit. We will first ask you some questions to make sure that you are well suited for the study. Your caregiver (e.g., family or friend you trust to make medical decisions with you) may be asked to also take part in the study if necessary. If you decide to participate then you will be "randomized" into one of the groups described below. Randomization means that you are put into a group by chance (like flipping a coin). There is no way to predict which group you will be assigned to. You will have an equal chance of being placed in either group. Neither you nor the study team can choose what group you will be in. You will be told which treatment you are to get. Group 1: If you are randomized to Group 1 you will be asked to have a brief interview with the study clinician to reflect on how your illness has affected your life. This brief interview will take about 20 minutes. Group 2: If you are randomized to Group 2, you will not have the brief interview. We will then ask you to complete some survey questions. To facilitate an ACP conversation with your primary outpatient doctor, with your permission, we will share what you discussed with your doctors and assist you with scheduling an outpatient doctor appointment. In total, this initial enrollment will take around 30-60 minutes. We will also contact you in about one month, three months, and six months after the initial enrollment to ask you some questions and offer ACP care navigation if needed. These questions will take about 30-45 minutes over the phone/Zoom (we will find time of your convenience). With your permission, we will record the initial enrollment and follow-up calls and store them in a safe hospital computer system. We will also check your medical records to see if you have new ACP documentation and other healthcare use.

**Payment:** You will receive a $48 gift card, or check (whichever you prefer) for participating in this study. If you choose to receive a check, we will obtain your social security number for IRS purposes and destroy the number when check is mailed out. It may take up to 8 weeks to mail the check to you.

**Confidentiality and Data Security**

We will remove your name and other personal information from the research data so that we cannot identify you with what you said. Only our study team will be able to match what you told us to you. We will store your information in a secure location. Only the study staff can review your study information unless you choose to share them with your clinical team.

**What are the risks associated with participation?**

We do not foresee any risk associated with participation in this study other than 30-60 extra minutes of your time during your stay in the ED/hospital and the potential risk of loss of confidentiality. We will ask you to think about your illness and care goals in the future, which may make you anxious. We can help you deal with these feelings if needed. Your participation is voluntary and you may stop at any time. Deciding not to participate will have no effect on your care.

**Questions:**

The PI can be contacted at the following 24 hours per day and 7 days per week:

Cell – 857-205-4947

Email – [kouchi@partners.org](mailto:kouchi@partners.org)

If you’d like to speak to someone not involved in this research about your rights as a research subject, or any concerns or complaints you may have about the research, contact the Partners Human Research Committee at 857-282-1900.

We are required by the Health Insurance Portability and Accountability Act (HIPAA) to protect the privacy of health information obtained for research. This is an abbreviated notice and does not describe all details of this requirement. During this study, identifiable information about you or your health will be collected and shared with the researchers conducting the research. In general, under federal law, identifiable health information is private. However, there are exceptions to this rule. In some cases, others may see your identifiable health information for purposes of research oversight, quality control, public health and safety, or law enforcement. We share your health information only when we must, and we ask anyone who receives it from us to protect your privacy.
